# Novel Functional eQTL-SNPs Associated With Susceptibility to *Mycoplasma pneumoniae* Pneumonia in Children

**DOI:** 10.3389/fpubh.2022.899045

**Published:** 2022-06-28

**Authors:** Yang Dong, Yanmin Gao, Cheng Luo, Nengshun Wu, Zhounan Cheng, Anni Qiu, Yan Zhou, Wendi Zhang, Minjie Chu, Qing Chang

**Affiliations:** ^1^Department of Epidemiology, School of Public Health, Nantong University, Nantong, China; ^2^Department of Pediatrics, No. 8 People's Hospital of Wuxi, Wuxi, China; ^3^Department of Laboratory Medicine, No. 8 People's Hospital of Wuxi, Wuxi, China

**Keywords:** *ACE*, *CD276*, *Mycoplasma pneumoniae* Pneumonia, single-nucleotide polymorphisms, susceptibility

## Abstract

**Background:**

The functional causal single-nucleotide polymorphisms (SNPs) associated with susceptibility to *Mycoplasma pneumoniae* Pneumonia (MPP) have scarcely been identified. In this study, we aimed to analyze the association between the functional expression quantitative trait locus (eQTL)-SNPs and the risk of MPP.

**Methods:**

First, we identified reported genes associated with MPP from the human disease database, MalaCards. After investigating multiple databases, we systematically selected seven functional eQTL-SNPs (rs2070874, rs360720, rs8032531, rs4316, rs4353, rs7258241, and rs2250656). Finally, the selected eQTL-SNPs were genotyped using the TaqMan genotyping technology, and compared between 100 children with MPP and 178 healthy controls.

**Results:**

We found that three eQTL-SNPs (rs8032531 in *CD276* and rs4316 and rs4353 in *ACE*) were significantly associated with susceptibility to MPP. Joint analysis of the three eQTL-SNPs revealed that the risk of MPP increased with an increase in the number of risk alleles present. Plasma protein expression levels of CD276 and ACE were distinctively higher in children with MPP than in healthy children (CD276: *P* < 0.001; ACE: *P* = 0.001).

**Conclusion:**

Functional eQTL-SNPs in *CD276* and *ACE* may affect the susceptibility to MPP. The risk of developing MPP is higher in patients harboring a greater number of unfavorable alleles of the aforementioned SNPs.

## Introduction

*Mycoplasma pneumoniae* (MP) is a major pathogen responsible for human respiratory infections. MP pneumonia (MPP) is the main cause of community-acquired pneumonia (CAP), which has an incidence as high as 40% (1). The prevalence of MPP is higher among children and is considered a significant clinical problem by pediatricians (2). The proportion of severe MPP cases has increased in recent years (3, 4). Moreover, MPP can affect multiple parts of the body and result in several extrapulmonary complications, which can be life-threatening in severe cases (5). In China, although the diagnostic and therapeutic strategies for the treatment of MPP have advanced with the rapid development of medical technology, the societal burden due to MPP has not been alleviated, possibly owing to some unnecessary early checking and an increase in non-pharmacological treatments (6). Therefore, it is imperative to examine the risk factors for MPP in children to aid in its prevention and treatment.

Although the causative agent of MPP is the MP pathogen, other factors such as sex, age, environment, and genetic background can also influence susceptibility to the disease (1). With the continuous advancement of human genomics research, many potential host genes associated with various diseases have been identified and studied, and MPP is no exception. For instance, some researchers have reported decreased expression of tumor necrosis factor-α-induced protein 8-like 2 (*TIPE2*) following MP infection, and subsequent *in vitro* studies have revealed that *TIPE2* may play a negative role in MP-triggered immune responses by inhibiting the mitogen-activated protein kinase (MAPK) signaling pathway (7). Chen et al. found that the expression of soluble *CD276* was significantly higher in bronchoalveolar lavage fluid (BALF) of children with MPP and may play an important role in the pathogenesis of MPP infection; thus, it may serve as a prognostic predictor or biomarker for MPP (8). Serum levels of CD276 have been demonstrated to not only differ significantly between children with MPP and healthy children but also be significantly higher in children with severe MPP than in children with mild MPP; therefore, testing levels of *CD276* may not only help in the early diagnosis of MPP but may also help judge the severity of the disease (9). Moreover, a high C-reactive protein/procalcitonin ratio could predict MP infection and improve its empirical management (10). Other similar studies have significantly promoted our understanding of the mechanism underlying MPP pathogenesis and the development of effective treatment strategies.

In addition to the abnormal expression of human genes, genetic variations (mainly caused by single-nucleotide polymorphisms, SNPs) in individual host genes are critical factors that determine the degree of individual response to MP infection (11). For instance, the interleukin-4 (*IL-4*) rs2227284 GG genotype in patients with MPP cases has been shown to induce a high burden of infection in children (12). In addition, angiotensin-converting enzyme (*ACE)* rs4340 and interleukin-6(*IL-6*) rs1800795 have been proven to be significantly associated with the risk of MPP in Chinese children (13). Moreover, the reported association of *ACE* rs4340 with the risk of MPP is consistent with the results of a previous study by Salnikova et al. (14). The development of advanced high-throughput genotyping technology in recent years has facilitated the identification of a large number of genetic variations, which has significantly promoted the screening, prevention, and treatment of MPP in high-risk individuals. Although some studies have discussed the individual susceptibility to MPP, these are predominantly based on single candidate genes and their tagging SNPs. They also lack a systematic evaluation of MPP-related genes and their functional causal SNPs.

The main aim of this study was to identify functional SNPs that may influence the risk of MPP, and to explore whether the corresponding proteins that these functional SNPs are located in are differentially expressed in the plasma of patients and controls. Thus, the following steps were undertaken. First, we identified the reported genes associated with MPP from the human disease database MalaCards and systematically screened these to identify the candidate functional causal SNPs. Subsequently, we conducted a case-control study (including 100 cases and 178 controls) to evaluate the association between these SNPs and susceptibility to MPP. Third, another case-control study was carried out to further explore whether the proteins targeted by significant SNPs were differentially expressed in the plasma of MPP patients and controls. The novelty of this study is that we not only focused on individual genes and their tagging SNPs but also systematically explored the entire collection of MPP-related genes listed in the human disease database MalaCards, with a broader scope and a greater likelihood of potentially meaningful findings. Furthermore, the SNP selection process combined with stringent selection criteria, including expression quantitative trait locus (eQTL) analysis, RegulomeDB scores, and minor allele frequency (MAF) filter, may increase the likelihood of obtaining functional causal SNPs associated with MPP susceptibility.

## Materials and Methods

### Study Design

We screened the preliminary data and subsequently conducted a case-control study to investigate the relationship between functional SNPs in genes implicated in MPP and susceptibility to the condition. At the screening stage, we identified MPP-associated genes using the human disease database MalaCards and filtered all SNPs located in those genes based on the 1,000 Human Genomes Project. This was followed by a series of screening tests to identify potential functional SNPs. In the subsequent population evaluation stage, we conducted a case-control study using TaqMan genotyping to determine the association between the identified functional SNPs and susceptibility to MPP.

### Sample Size Evaluation

Power and Sample Size Calculation (PS) software (version 3.1.2) was used to calculate the sample size. Setting a two-sided significance level alpha of 0.05, a statistical power of 0.80, a variant allele frequency of 0.41 (the maximum value of the MAF corresponding to the eQTL-SNPs selected in this study was 0.4126) and an effect OR of 2.0, the results indicated that at least 132 cases and 132 controls were required.

### Study Population

This study included 100 children diagnosed with MPP (case group) who were hospitalized at the Department of Pediatrics, No. 8 People's Hospital of Wuxi, from October 2019 to July 2021. The control group consisted of 178 healthy children who had undergone check-ups in the child health clinic during the same period and were matched for sex and age. Peripheral blood samples of all children were collected after obtaining written consent from their families. The study was reviewed and approved by the Ethics Committee of No.8 People's Hospital of Wuxi.

#### Diagnostic Criteria

All selected cases met the following two conditions: (1) the diagnostic criteria for pneumonia including fever, cough, dyspnea, pulmonary rales, and other respiratory manifestations, accompanied by unilateral or abnormal changes in chest imaging, suggesting parenchymal infiltration of the lungs and (2) criteria for MP infection including positive MP-DNA in the bronchoalveolar lavage fluid (BALF) through PCR fluorescent probe assay and positive MP antibody. Patients with metabolic diseases, benign or malignant tumors, autoimmune diseases, and other respiratory pathogen infections were excluded from the study.

### Selection of MPP-Related Genes and Functional eQTL-SNPs

**Figure 1:**
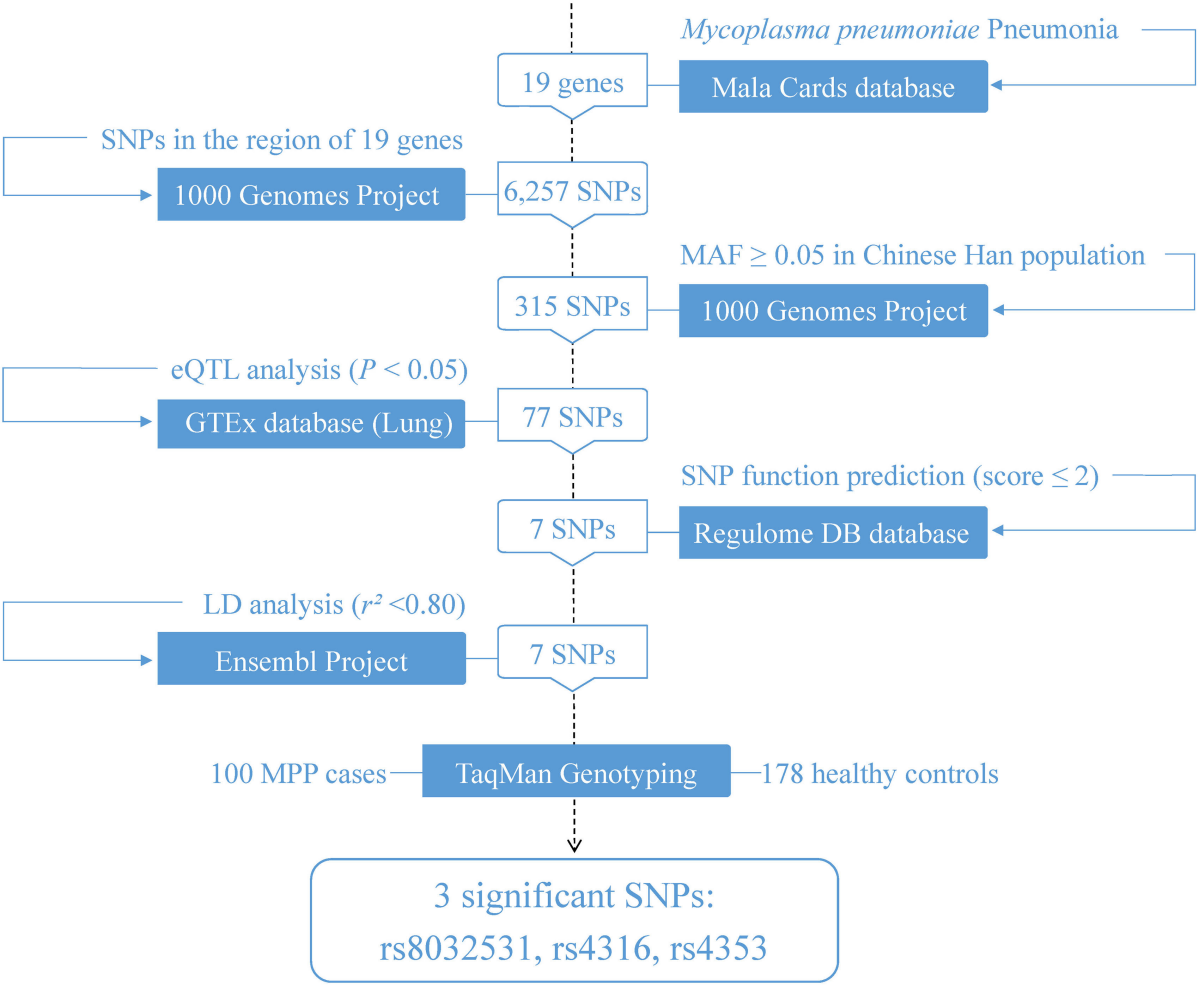
Flowchart of the study design. eQTL, expression quantitative trait locus; SNP, single-nucleotide polymorphism; LD, linkage disequilibrium; MAF, minor allele frequency.

First, 19 genes related to MPP were identified using the keyword “*Mycoplasma pneumoniae* Pneumonia” from the human disease database MalaCards (15) (https://www.malacards.org/) in June 2021. Subsequently, based on the 1,000 Genome Project, common SNPs (MAF ≥ 0.05) in the Han Chinese population in Beijing were screened in the regions where the 19 genes were located. Next, expression quantitative trait locus (eQTL) analysis in lung tissues was performed using the GTEx database (http://www.gtexportal.org/home/) to screen for candidate functional eQTL-SNPs. A total of 77 eQTL-SNPs associated with a differential expression of MPP-related genes in the lung tissue were identified. Following this, the potential functions of the selected SNPs were predicted using RegulomeDB (16) (http://regulomedb.org/; RegulomeDB score ≤ 2). These functional eQTL-SNPs were further filtered using linkage disequilibrium analysis (LD) through the Ensembl Project (http://www.ensembl.org/), retaining one when SNPs with high LD (*r*^2^ ≥ 0.8). Eventually, seven functional eQTL-SNPs passed the screening and were included in the next stage of genotyping (Figure 1).

### Genotyping

Genomic DNA was extracted from peripheral blood using a DNA extraction kit (Qiagen, Hilden, Germany), and genotyping was performed using the TaqMan allelic discrimination protocol on the Applied Biosystems™ QuantStudio™ 5 FAST Real-Time PCR System (TaqMan; Applied Biosystems, United States). All TaqMan probes and primers were designed and manufactured by BioSteed BioTechnologies, Nanjing, China. The genotyping success rate was more than 99%.

### Measurement of Plasma Protein Levels of CD276 and ACE

Collected plasma samples were stored at −80°C, and the protein levels of CD276 and ACE were measured using enzyme-linked immunosorbent assay (ELISA) kits (MEIMIAN, Jiangsu, China) according to the manufacturer's instructions. Optical density values were read at 450 nm using a microplate reader (Sunrise, Tecan, Austria). The plasma protein concentrations of CD276 and ACE were obtained separately from the standard curves generated by diluting the known molecular concentrations provided in the kits.

### Statistical Analysis

The two-sided chi-square test and Student's *t*-test were used for categorical and continuous variables, respectively, to assess the differences in demographic characteristics between the cases and controls. We performed logistic regression analysis to evaluate the eQTL-SNPs that correlated with the risk of MPP using odds ratios (ORs) and 95% confidence intervals (CIs) after adjusting for age and sex. Furthermore, logistic regression analysis was conducted after adjusting for age and sex to estimate the association of the candidate eQTL-SNPs with MPP susceptibility via OR values and 95% CI. Generally, for each eQTL-SNP, we assigned those carrying no minor allele to the wild-type homozygote (code 0), those carrying one minor allele to the heterozygote (code 1), and those carrying two minor alleles to the variant homozygote (code 2). Next, we assessed the association in different genetic models: heterozygote (co-dominant) model (1 vs. 0), homozygote (co-dominant) model (2 vs. 0), dominant model (1 + 2 vs. 0), recessive model (2 vs. 1 + 0). For the additive model, testing was designed specifically to reveal associations that depended additively on the minor allele; that is, subjects carrying two minor alleles (as compared with those carrying no minor allele) were twice as likely to affect the outcome in a certain direction as subjects carrying one minor allele (as compared with those carrying no minor allele). In addition, the *P* value corresponding to the model chi-squared value in each regression analysis was >0.05, indicating a good model fit. We included potential confounding variables (e.g., sex and age) in the regression analysis model to produce adjusted OR to control for the effect of confounding factors. Differences in the levels of the relevant proteins in the plasma of patients and controls were analyzed using the Student's *t*-test. *P*-values < 0.05 were considered statistically significant. The statistical analyses were performed using Stata version 15.0 (StataCorp, College Station, TX, United States), SPSS version 20.0 (IBM Corp., Armonk, NY, United States), and R version 3.6.2 software (R Foundation for Statistical Computing, Vienna, Austria), as needed.

## Results

### Characteristics of the Participants

The basic characteristics of the 100 children with MPP and 178 healthy controls included in this study are shown in Table 1. Overall, the sex and age ratio between the case and control groups were comparable, and 88% of the patients tested positive for IgM, 73% for IgG, and 60% for both.

**Table 1:** Characteristics of the subjects enrolled in this study.

**Variables**	**Case (*n* = 100)**	**Control (*n* = 178)**	***P***
**Age**			0.070
≤ 4	56 (56.00%)	120 (67.42%)	
> 4	44 (44.00%)	58 (32.58%)	
**Gender**, ***n*** **(100%)**			0.269
Male	51 (51.00%)	103 (57.87%)	
Female	49 (49.00%)	75 (42.13%)	
**Antibody detection**			
IgM (+)	88 (88.00%)		
IgG (+)	73 (73.00%)		
IgM (+) & IgG (+)	60 (60.00%)		

### Associations Between the Seven Candidate eQTL-SNPs and MPP Risk

**Figure 2:**
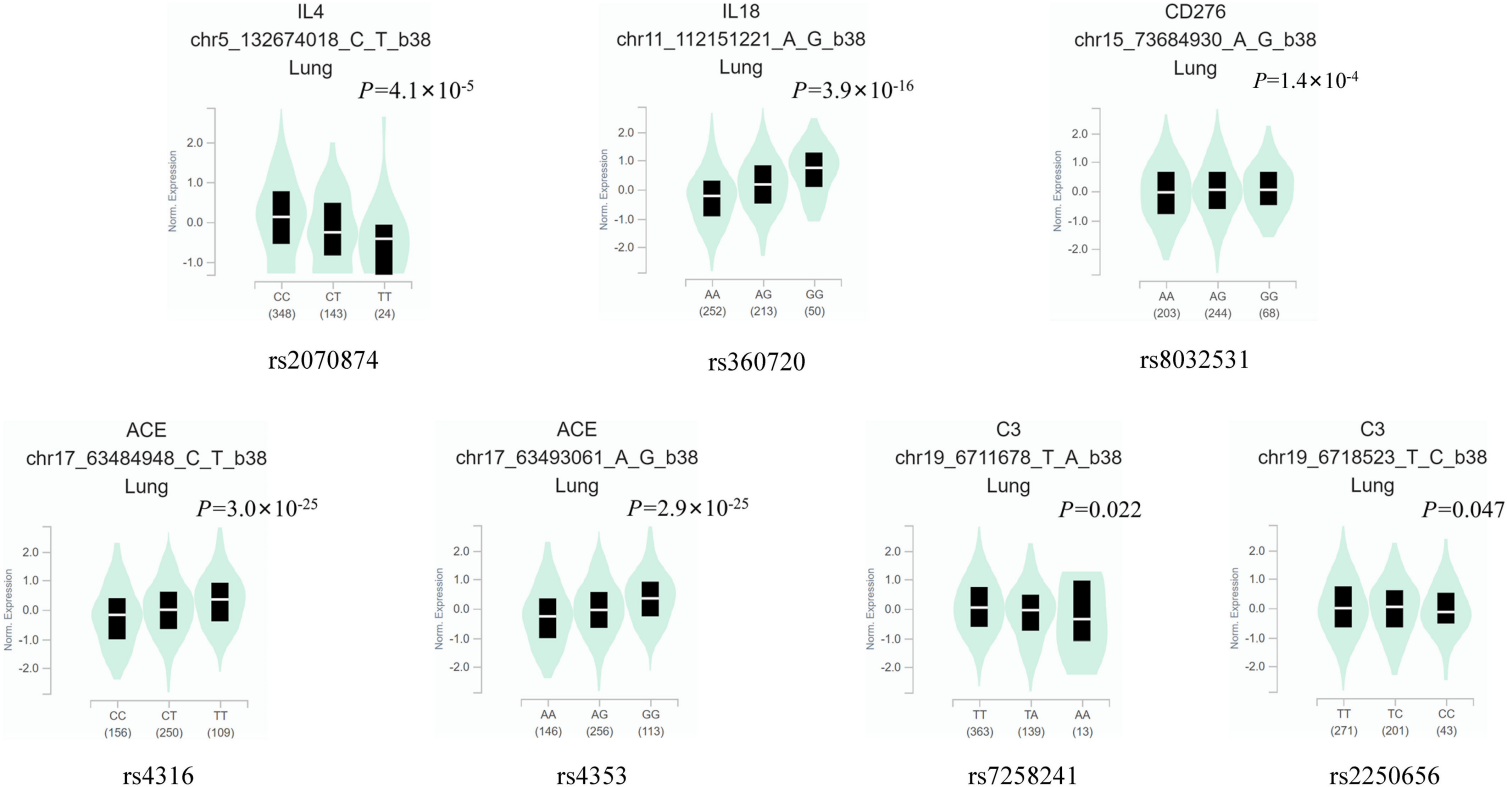
The eQTL direction of the seven eQTL-SNPs in the GTEx database. eQTL, expression quantitative trait locus; SNP, single-nucleotide polymorphism.

Of the 19 MPP-related genes available in the MalaCards database (Table 2), seven candidate functional eQTL-SNPs were selected through a series of screening tests. Scores for 19 genes were provided by the MalaCards Composite Related Gene Score (MCRGS), details of which can be found on the MalaCards database website. This is a correlation score assigned by MalaCards to each disease-gene association, which reflects the degree of correlation between them. Information on the seven eQTL-SNPs is presented in Table 3, and their eQTL direction in the GTEx database is shown in Figure 2. Among these, the rs8032531 variant G allele in *CD276* (OR = 1.67, 95% CI = 1.12–2.49, *P* = 0.012), rs4316 variant T allele in *ACE* (OR = 2.05, 95% CI = 1.40–2.99, *P* < 0.001), and rs4353 variant G allele in *ACE* (OR = 2.26, 95% CI = 1.55–3.29, *P* < 0.001) were significantly associated with an increased risk of MPP. The association between the three eQTL-SNPs and susceptibility to MPP under different genetic models is summarized in Table 4.

**Table 2:** 19 genes related to MPP.

**Number**	**Gene**	**Description**	**Chr**	**Category**	**Score^a^**
1	CRP	C-Reactive Protein	Chr1: 159,682,079–159,684,379	Protein Coding	14.08
2	IL17A	Interleukin 17A	Chr6: 52,051,173–52,055,436	Protein Coding	13.63
3	TNF	Tumor Necrosis Factor	Chr6: 31,543,342–31,546,113	Protein Coding	13.59
4	IL10	Interleukin 10	Chr1: 206,940,947–206,945,839	Protein Coding	13.39
5	IL18	Interleukin 18	Chr11: 112,013,983–112,034,817	Protein Coding	13.26
6	IL6	Interleukin 6	Chr7: 22,766,819–22,771,617	Protein Coding	13.25
7	IFNG	Interferon Gamma	Chr12: 68,548,548–68,553,520	Protein Coding	12.94
8	IL4	Interleukin 4	Chr5: 132,009,681–132,018,370	Protein Coding	12.91
9	IL13	Interleukin 13	Chr5: 131,992,214–131,996,802	Protein Coding	12.44
10	TLR2	Toll Like Receptor 2	Chr4: 154,605,432–154,627,412	Protein Coding	12.22
11	CD276	CD 276 Molecule	Chr15: 73,976,724–74,006,855	Protein Coding	12.21
12	C3	Complement C 3	Chr19: 6,677,715–6,720,661	Protein Coding	11.65
13	F3	Coagulation Factor III, Tissue Factor	Chr1: 94,994,729–95,007,315	Protein Coding	11.58
14	IL23A	Interleukin 23 Subunit Alpha	Chr12: 56,732,668–56,734,194	Protein Coding	11.47
15	CSF2	Colony Stimulating Factor 2	Chr5: 131,409,482–131,411,863	Protein Coding	11.46
16	ACE	Angiotensin I Converting Enzyme	Chr17: 61,554,422–61,575,734	Protein Coding	11.35
17	MUC1	Mucin 1, Cell Surface Associated	Chr1: 155,158,300–155,162,706	Protein Coding	11.3
18	SFTPD	Surfactant Protein D	Chr10: 81,697,496–81,708,861	Protein Coding	11.23
19	CYP1A1	Cytochrome P450 Family 1 Subfamily A Member 1	Chr15: 75,011,883–75,017,869	Protein Coding	10.8

a *Score is a weighted sum of the scores of the associations between MPP and the listed gene in multiple databases collected in the MalaCards database*.

**Table 3:** Association between the 7 SNPs and MPP risk.

**Gene**	**SNP**	**Position**	**Location**	**Alleles^a^**	**Cases^b^**	**Controls^b^**	**MAF^c^ (case/control)**	**OR(95%CI)^d^**	***P*** **^d^**
IL4	rs2070874	132009710	Exon	T/C	65/31/4	117/55/6	0.20/0.19	1.10 (0.70–1.73)	0.672
IL18	rs360720	112021944	Intron	T/C	76/23/1	143/34/1	0.13/0.10	1.31 (0.74–2.30)	0.351
CD276	rs8032531	73977271	Intron	A/G	23/64/13	71/89/18	0.45/0.35	1.67 (1.12–2.49)	0.012
ACE	rs4316	61562309	Intron	C/T	26/53/21	82/79/17	0.48/0.32	2.05 (1.40–2.99)	<0.001
ACE	rs4353	61570422	Intron	A/G	19/51/30	72/84/22	0.56/0.36	2.26 (1.55–3.29)	<0.001
C3	rs7258241	6711689	Intron	T/A	50/44/6	85/69/24	0.28/0.33	0.79 (0.54–1.15)	0.225
C3	rs2250656	6718534	Intron	A/G	65/33/2	97/73/8	0.19/0.25	0.66 (0.42–1.04)	0.075

a *Major/minor allele. Major allele was used as reference allele*.

b *Wild-type homozygote/heterozygote/variant homozygote*.

c *MAF: minor allele frequency*.

d *Logistic regression analysis adjusted for age and sex in the additive model. OR, odds ratio; CI, confidence interval*.

**Table 4:** Association of 3 significant SNPs with MPP risk under different genetic models.

**SNP**	**Genotypes**	**Cases, *n* (100%)**	**Controls, *n* (100%)**	**Adjusted OR (95% CI)^a^**	***P*** ^a^
rs8032531	AA	23 (23.00%)	71 (39.89%)	1 (ref.)	
	AG	64 (64.00%)	89 (50.00%)	2.28 (1.28–4.05)	0.005
	GG	13 (13.00%)	18 (10.11%)	2.24 (0.94–5.31)	0.068
	Dominant model			1.31 (0.61–2.84)	0.488
	Recessive model			2.27 (1.30–3.98)	0.004
	Additive model			1.67 (1.12–2.49)	0.012
rs4316	TT	26 (26.00%)	82 (46.07%)	1 (ref.)	
	TC	53 (53.00%)	79 (44.38%)	2.11 (1.19–3.72)	0.010
	CC	21 (21.00%)	17 (9.55%)	4.12 (1.87–9.09)	<0.001
	Dominant model			2.67 (1.32–5.43)	0.006
	Recessive model			2.45 (1.43–4.21)	0.001
	Additive model			2.05 (1.40–2.99)	<0.001
rs4353	GG	19 (19.00%)	72 (40.45%)	1 (ref.)	
	GA	51 (51.00%)	84 (47.19%)	2.37 (1.28–4.41)	0.006
	AA	30 (30.00%)	22 (12.36%)	5.08 (2.39–10.80)	<0.001
	Dominant model			2.93 (1.57–5.48)	0.001
	Recessive model			2.95 (1.64–5.32)	<0.001
	Additive model			2.26 (1.55–3.29)	<0.001

a *Logistic regression analysis adjusted for age and sex*.

### Joint Analysis of the Three Significant eQTL-SNPs on the Risk of Developing MPP

**Figure 3:**
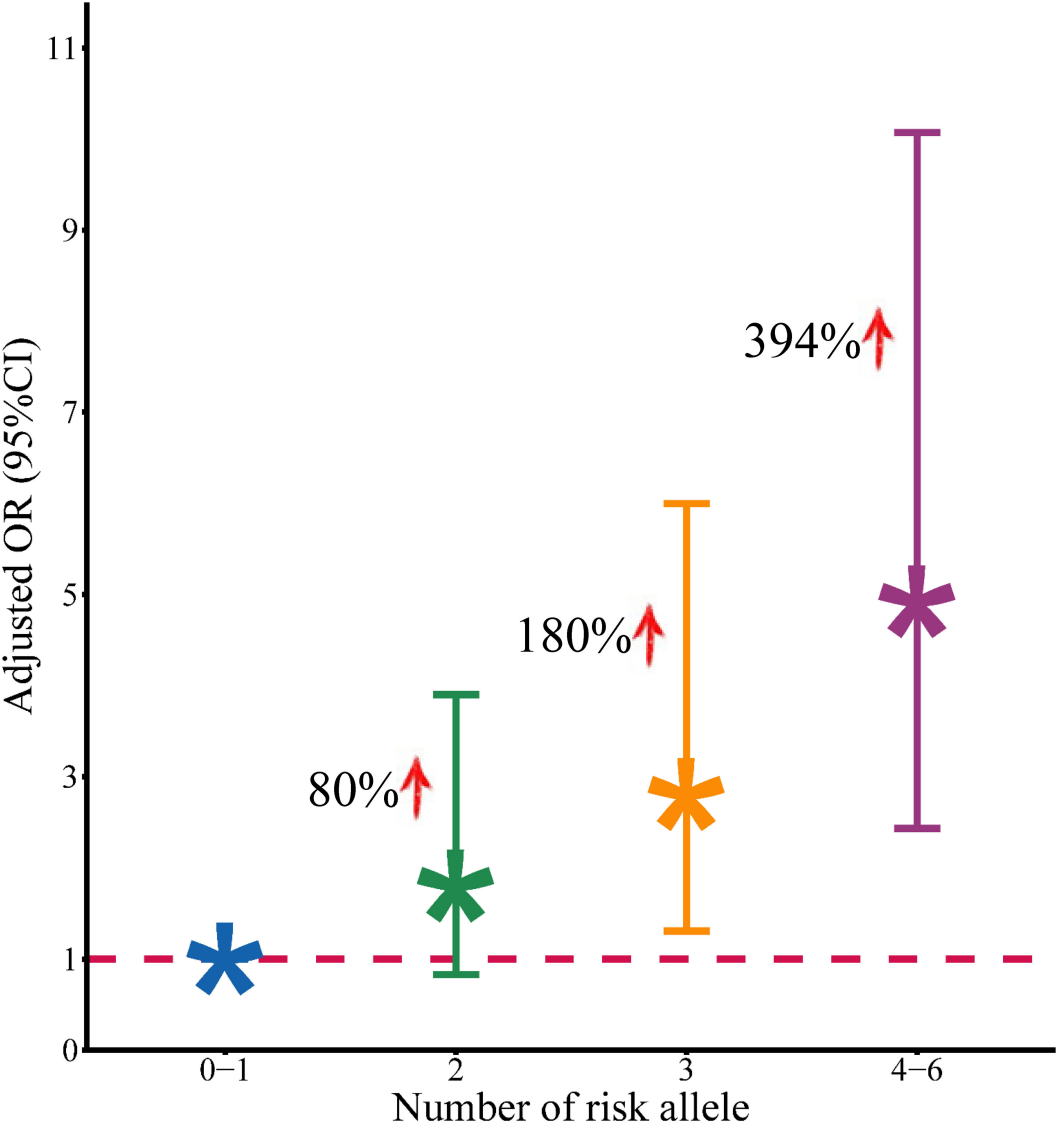
Association between the number of risk alleles of positive SNPs and the risk of development of MPP.

Based on the abovementioned results, we performed a joint analysis on the three significant eQTL-SNPs, including rs8032531 (A > G), rs4316 (T > C), and rs4353 (G > A). As presented in Table 5, the risk of developing MPP was higher in patients with a higher number of risk alleles of the above three significant eQTL-SNPs. Compared with individuals with 0–1 risk alleles, individuals with 2, 3, or 4–6 risk alleles had an increased risk of developing MPP by 80, 180, and 394%, respectively (Figure 3).

**Table 5:** Joint effect of 3 significant SNPs on MPP risk.

**Number of risk allele^a^**	**Case (*n* = 100) *n* (%)**	**Control (*n* = 178) *n* (%)**	**Adjusted OR (95% CI)^b^**	***P*** **^b^**
0–1	19 (19.00%)	73 (41.00%)	1 (ref)	–
2	18 (18.00%)	40 (22.50%)	1.80 (0.83–3.90)	0.137
3	23 (23.00%)	33 (18.50%)	2.80 (1.31–6.00)	0.008
4–6	40 (40.00%)	32 (18.00%)	4.94 (2.43–10.07)	<0.001
Trend test				<0.001

a *rs8032531-G, rs4316-C and rs4353-A alleles were assumed as risk alleles*.

b *The reference group was those with “0–1” risk allele. Adjusted for age and sex*.

### Differences in the Plasma Levels of the Target Proteins

**Figure 4:**
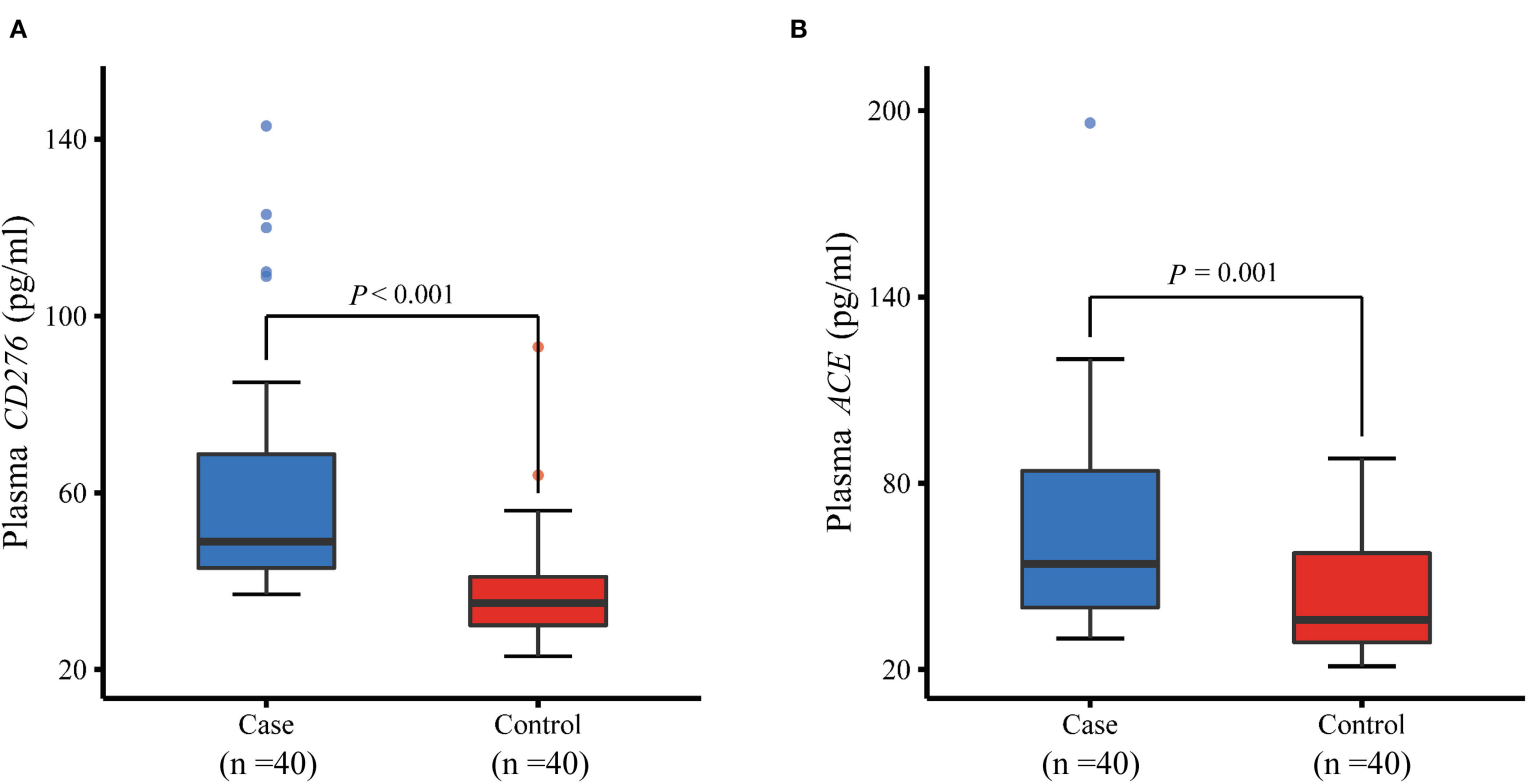
Comparison of plasma protein levels of CD276 and ACE between MPP cases and controls measured by ELISA. **(A)** Plasma protein level of CD276 was significantly higher in children with MPP than in healthy controls (*P* < 0.001). **(B)** Plasma protein level of ACE increased considerably in children with MPP compared with healthy controls (*P* = 0.001). ELISA, enzyme-linked immunosorbent assay; MPP, *Mycoplasma pneumoniae* Pneumonia.

**Figure 5:**
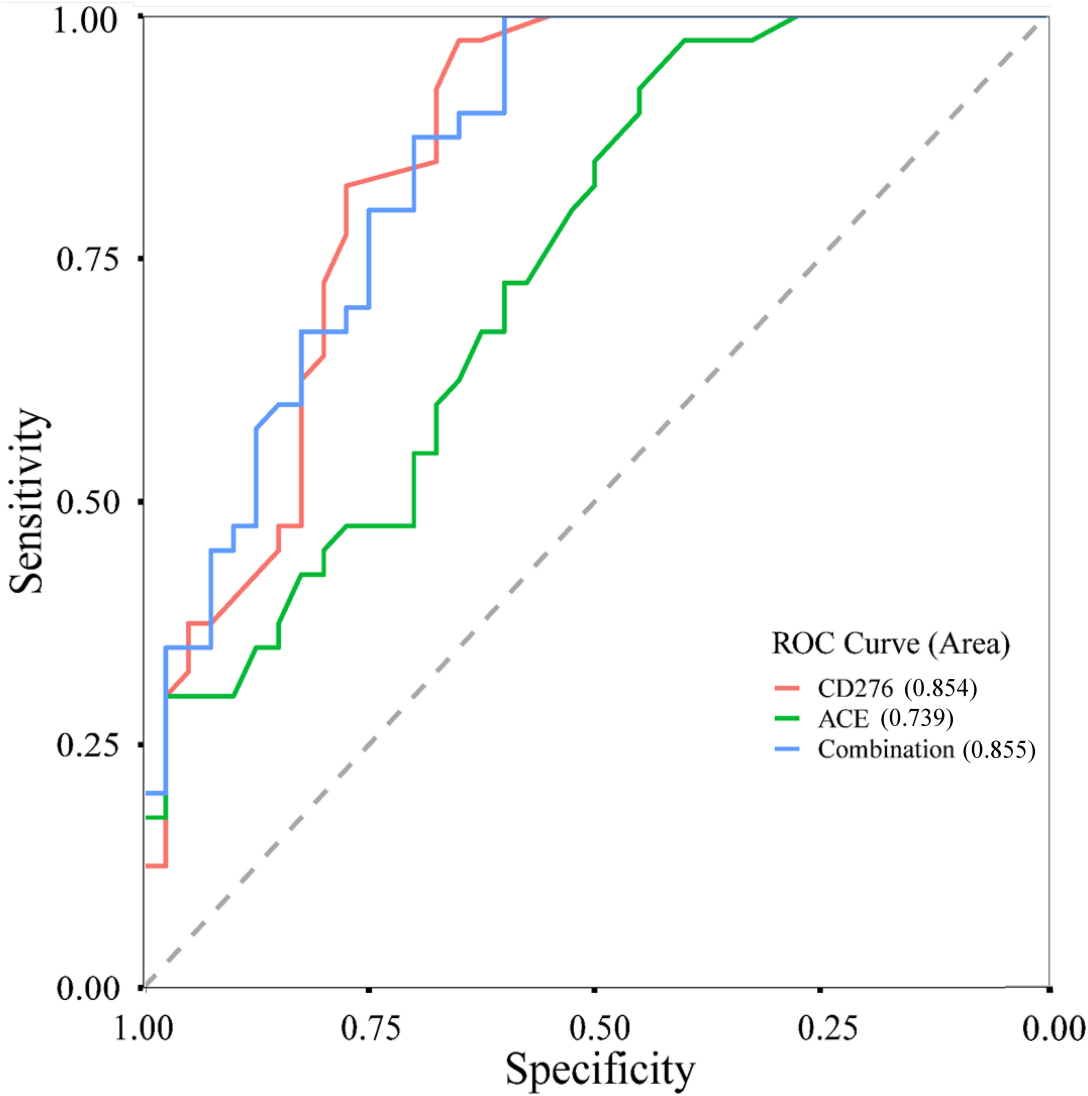
ROC curves of CD276, ACE and their combined levels in children with MPP and healthy controls. ROC, receiver operating characteristic; MPP, *Mycoplasma pneumoniae* Pneumonia.

The genes *CD276* and *ACE* were targeted by the aforementioned three significant eQTL-SNPs. Furthermore, we measured the plasma protein expression levels of CD276 and ACE in 40 patients with MPP and 40 healthy controls. As illustrated in Figure 4, the levels of both proteins were significantly higher in the patients than in the controls (CD276: *P* < 0.001; ACE: *P* = 0.001). We used the protein levels of the cases and controls (*n* = 40 each) to calculate the receiver operating characteristic (ROC) curves and the area under the ROC curves (AUC): the ROC curves showed a clear distinction between them (Figure 5). The sensitivity and specificity of CD276 were 82.5 and 77.5%, respectively (AUC: 0.854, 95% CI: 0.769–0.938, *P* < 0.001). Similarly, the sensitivity and specificity of ACE were 72.5 and 60.0%, respectively (AUC: 0.739, 95% CI: 0.632–0.846, *P* < 0.001).

## Discussion

MPP is a global public health problem in children; however, few relevant studies have considered the host point of view on genetic variations. Therefore, we conducted a case-control study on 100 children with MPP and 178 healthy children, and systematically explored the relationship between the functional eQTL-SNPs of 19 MPP-related genes and susceptibility to MPP. Among the seven pre-selected functional eQTL-SNPs, we found that rs8032531 in *CD276* and rs4316 and rs4353 in *ACE* were significantly associated with susceptibility to MPP. The risk of developing MPP was higher in children harboring a greater number of risk alleles. Moreover, the plasma levels of CD276 and ACE were significantly higher in the patients than in healthy controls.

*CD276*, also known as B7 homolog 3 protein (*B7-H3*), is a newly identified cell surface molecule from the B7 family. It is widely expressed in various organs and tissues and has a co-stimulatory as well as a co-inhibitory function in T cells (17). *CD276* plays an important role in the regulation of both autoimmunity and inflammation; therefore, it has been extensively studied as a promising therapeutic target for immune diseases in recent years (18). Chen et al. reported that *CD276* was involved in the enhancement of the formation of associated immune complexes and the activation of the NF-κB p65 and MAPK p38 pathways downstream of Toll-like receptor 2 (TLR2) in the brains of *Streptococcus pneumoniae*-infected mice, thereby leading to the amplification of the inflammatory response and exacerbation of brain injury. Moreover, some studies demonstrated that blocking NF-κB p65 and/or MAPK p38 and their specific inhibitors significantly attenuated the inflammatory response amplified by *CD276* and reduced the production of pro-inflammatory cytokines and chemokines, and hence can be considered a potential therapeutic strategy to ameliorate the inflammatory damage caused by *Streptococcus pneumoniae* infections (19). A majority of studies have reported that the action of *CD276* is dependent on TLR2 pathway signaling; however, this is not always the case. A study reported that although TLR2 deletion blocked the pathogenesis of asthma; it did not terminate the pro-inflammatory effect of *CD276* (20). This implies that the pro-inflammatory effects of *CD276* are not restricted to TLR2-dependent mechanisms. In addition, in recent years, *CD276* has been found to be involved in the excessive immune response caused by MP. Soluble *CD276* is overexpressed and is positively correlated with the inflammatory cytokines IFN-γ and GM-CSF in the plasma of children with MPP, thereby enhancing the inflammatory response (21). In conclusion, *CD276* is known to participate in the inflammatory response to MPP and is a valid target gene that can be used to explore the mechanisms underlying the development of MPP.

*ACE* is a dicarboxypeptidase that was initially thought to play an important role in regulating blood pressure. Upon identification of its multiple peptide substrates and broad tissue distribution, *ACE* was found to be involved in a variety of biological functions, most notably in the immune system (22). Numerous reports have suggested that the expression of *ACE* is upregulated in several inflammatory diseases. Moreover, a growing body of evidence suggests that *ACE* overexpression is accompanied by an increase in immune response to inflammatory stimuli (23). Neutrophils when accompanied by high *ACE* expression are more effective and significantly increase superoxide production, resulting in higher levels of the pro-inflammatory cytokine IL-1β and enhanced immune resistance (24). Moreover, overexpression of *ACE* in myeloid cells enhances the immune response against tumors, infections, Alzheimer's disease, and atherosclerosis in mice models (25).

Over the past 2 years, coronavirus disease 2019 (COVID-19) has affected people globally (26, 27). It is an acute respiratory disease caused by severe acute respiratory syndrome coronavirus 2 (SARS-CoV-2), and *ACE2* is an important receptor involved in the entry of SARS-CoV-2 into cells. Studies have shown that an imbalance in the interaction between *ACE* and *ACE2* may be one of the chief mechanisms underlying COVID-19-related lung injury. Therefore, testing patients for *ACE* gene polymorphisms may help in disease prevention and targeted treatment (28). It is, therefore, plausible that *ACE* influences the development of MPP in terms of immune function.

One of the strengths of our study is that we comprehensively explored reported genes related to MPP listed in the MalaCards database and studied the association between functional SNPs on those genes and susceptibility to MPP in children. Additionally, we combined multiple databases such as the 1,000 Genome Project, GTEx, and RegulomeDB while selecting the functional SNPs based on a series of stringent principles, which significantly increased the possibility of identifying causal SNPs associated with MPP susceptibility. To the best of our knowledge, this is the first report of a novel functional eQTL-SNP on *CD276* associated with susceptibility to MPP. Although SNPs on *ACE* have previously been reported to be associated with MPP, the functions of the relevant SNPs have not been well defined. Herein, we identified novel functional eQTL-SNPs on *ACE* associated with the risk of MPP.

There were some limitations to this study. For example, although the case sample size calculated was expected to be 132, up to the time of genotyping and other related experiments, we had only been able to collect 100 cases and 178 controls. We will continue to arrange for the collection of samples to expand our sample database afterward. Moreover, the chosen parameters for the sample size evaluation were relatively broad. Therefore, even if we achieve the expected number of cases, further studies with samples from different hospitals in different areas will be needed to validate our results. In addition, although the screening process in this study was stringent, such as the use of MAF filter, eQTL analysis, and RegulomeDB scores, this may have caused some significant SNPs to be filtered out at the screening stage, which could potentially lead to more false negatives. It is expected that a more effective screening strategy will be available to avoid the problem of false negatives in the future.

## Conclusion

In conclusion, we identified three eQTL-SNPs (rs8032531 in *CD276* and rs4316 and rs4353 in *ACE*) that were significantly associated with susceptibility to MPP. The risk of developing MPP was higher in subjects harboring a greater number of unfavorable alleles of the aforementioned SNPs. Plasma protein expression levels of CD276 and ACE were distinctively higher in children with MPP than in healthy children.

## Data Availability Statement

The raw data supporting the conclusions of this article will be made available by the authors, without undue reservation.

## Ethics Statement

The studies involving human participants was reviewed and approved by the Ethics Committee of No. 8 People's Hospital of Wuxi. Written informed consent to participate in this study was provided by the participants' legal guardian/next of kin.

## Author Contributions

YD: conceptualization, investigation, writing—original draft, and writing—review and editing. CL: conceptualization, investigation, resources, and writing—original draft. YG: conceptualization and writing—original draft. NW: formal analysis and software. ZC: data curation and investigation. AQ: data curation, formal analysis, and investigation. YZ: data curation. WZ: formal analysis. MC: funding acquisition, investigation, methodology, supervision, validation, and visualization. QC: funding acquisition, methodology, resources, and visualization. All authors contributed to the article and approved the submitted version.

## Funding

This work was supported by the Wuxi City Key Clinical Specialty (Occupational Disease) Construction Plan and the Scientific Research Project of the Wuxi Commission of Health (Z201906). The funding sources had no role to play in the study design, the collection and interpretation of the data, writing of the report, or decision to submit this paper for publication.

## Conflict of Interest

The authors declare that the research was conducted in the absence of any commercial or financial relationships that could be construed as a potential conflict of interest.

## Publisher's Note

All claims expressed in this article are solely those of the authors and do not necessarily represent those of their affiliated organizations, or those of the publisher, the editors and the reviewers. Any product that may be evaluated in this article, or claim that may be made by its manufacturer, is not guaranteed or endorsed by the publisher.
